# Do Students With Different Majors Have Different Personality Traits? Evidence From Two Chinese Agricultural Universities

**DOI:** 10.3389/fpsyg.2021.641333

**Published:** 2021-04-29

**Authors:** Xicheng Wen, Yuhui Zhao, Yucheng T. Yang, Shiwei Wang, Xinyu Cao

**Affiliations:** ^1^College of Horticulture, Nanjing Agricultural University, Nanjing, China; ^2^College of Public Administration, Nanjing Agricultural University, Nanjing, China; ^3^College of Engineering, Nanjing Agricultural University, Nanjing, China; ^4^Department of Molecular Biophysics and Biochemistry, Yale University, New Haven, CT, United States; ^5^Psychological Counseling Center, Nanjing Agricultural University, Nanjing, China; ^6^College of Foreign Studies, Nanjing Agriculture University, Nanjing, China

**Keywords:** agriculture-related majors, agricultural education, personality traits, college students, China

## Abstract

This paper explores whether a Student’s choice of major leads to certain personality traits and the reasons for this phenomenon. Specifically, we look at evidence from two Chinese universities, both of which specialize in agricultural studies. Using the Sixteen Personality Factor (16PF) questionnaire and the Neuroticism Extraversion Openness Five-Factor Inventory (NEO-FFI) questionnaire, we collected data from two groups of students: those who study agriculture-related majors (ARM), and those who study non-agriculture-related majors (NARM). The surveys all showed no significant change in personality traits during Students’ freshman year. However, after 3 years of university study, significant personality trait changes were noted between seniors in the ARM and NARM groups. Whereas ARM seniors tended to be socially shy and lower in communicative competence, NARM seniors were better at expressing themselves and communicating with others. Although a Student’s choice of profession has an influence on their personality traits, it is not the only factor. The differences between ARM and NARM training models and curricula are also undoubtedly significant. Moreover, the bias against ARM in Chinese society further magnifies the differences in personality traits among students with different majors.

## Introduction

Personality traits are defined as the relatively enduring and stable patterns of thought, feeling, and behavior that distinguish an individual from other individuals (Per and Beyoğlu, 2011). Personality traits can explain why certain behaviors occur, because an individual’s values and preferences reflect their traits, and those traits influence their actions (De Raad and Schouwenburg, 1996; Gentry William et al., 2007; Van Bragt et al., 2011; Wang et al., 2016; Navidinia et al., 2017). Although personality traits are characterized by persistence and stability, it is no longer controversial that personality traits can (be) change(d). What is at issue is when these traits stabilize and why they change.

Research suggests that personality traits only exhibit stability at certain age intervals. Personality traits change from late childhood (around age 10) to old age (starting at age 60) as one moves through various life stages. These stages can include schooling, employment, marriage, etc. (Twenge, 2009). Although the *hard plaster hypothesis* suggests that most personality changes occur before the age of thirty and gradually stabilize after that (Srivastava et al., 2003), Specht et al. (2011) argue that there is no single age group in which personality traits reach a stable peak; rather, personality changes occur over the course of one’s life. Moreover, there are differences between childhood and subsequent years. As individuals grow older, ties outside of the family gradually increase, and specific social environments come to replace the family context as the main factor influencing changes in personality traits (Soto et al., 2011).

The higher education stage is undoubtedly the most critical time period for individuals, as they are transitioning from adolescence to young adulthood. This stage is crucial for the development of personality traits. Students who leave home become more assertive during college, begin to learn to live independently, and become more emotionally stable (Robins et al., 2005; Roberts et al., 2006). The social environment at university may further influence personality traits; for example, individuals who interact more with others during their studies show an increase in enthusiasm and extroversion (Oreopoulos and Salvanes, 2009).

The influence of college on the formation of personality traits is undeniable, but whether students in different majors develop different personality traits is another question to consider. Before entering college, students do indeed choose distinct majors according to their interests and plans. Still, the teaching style, teaching environment, and even the people students come into contact with inside and outside of school differ greatly. As students progress through their studies, their personality traits are continually reshaped by an evolving understanding of their profession and the demands it places on them (Moreau and Leathwood, 2006).

There is no absolute good or bad personality trait, what matters is whether it is suitable for study, work, or social interaction (Rudolph et al., 2017). Every major is a foundation for specific career paths (Birnbaum and Umbach, 2001). However, modern higher education is no longer just about students acquiring professional skills; the ability to integrate into a team and society are also an important focus (Barth et al., 2007; Jackson, 2015). Taking this into account, it makes sense to explore the differences in personality traits among students of different majors. One of the primary purposes of contemporary research in educational psychology is to help students find suitable employment beyond their majors (Vedel, 2016). The two main lines of research related to this are Students’ choice of majors, and the consequences stemming from that choice. This paper focuses on the latter, including the differences in Students’ personality traits after completing different majors and the reasons for this difference.

This paper analyzes the differences in personality traits between students with agriculture-related majors (ARM) and students with non-agriculture-related majors (NARM) and how their distinct educational paths shape those differences. An important factor is the unique nature of agricultural education in China. On the one hand, the Chinese government places great emphasis on the importance of agricultural education; after all, up to now, the agricultural population still accounts for nearly half of China’s population. On the other hand, due to historical and prejudicial influences, ARM majors have not been popular in China for a long time. By analyzing the differences in personality traits among these two groups of students, and by exploring the reasons for those differences, we can provide some ideas that will aid future education reform.

## The Present Study

### Personality Trait Differences Across Academic Majors

Students in different majors tend to have distinct personality traits. For example, medical majors scored highest in extraversion and agreeableness (Lievens et al., 2002), while business majors scored higher in emotional stability and assertiveness (Lounsbury et al., 2009). Some studies have suggested that differences in these traits naturally lead students to choose different majors (Digman and Takemoto-Chock, 1981; Downey et al., 2011). However, the preferences reflected by personality traits do not fully influence the choice of college majors.

First, students often select popular majors based not on their interests, but rather on a combination of factors other than the major, such as employment and salary. This is especially true in East Asia (Xu, 2016; Denice, 2020). Second, each major requires students to have a particular set of personality traits. Several studies have pointed out that students with certain specific traits (in addition to those generally conducive to academics, such as focus and conscientiousness) are likely to do better in their majors (Komarraju and Karau, 2005; Komarraju et al., 2009). In other words, students can achieve better educational success when their personality traits are matched with the traits most appropriate for their major. Students who do not match the traits associated with their major, on the other hand, have a hard time standing out in a competitive college environment.

Therefore, students entering college may have some personality traits that match their professional traits; however, this “matching” should not be overstated in the case of freshmen. A university education develops Students’ professional skills while simultaneously shaping certain personality traits to match their area of study (Vedel, 2016). After 4 years of university education, the personality traits of students tend to change.

### Bias in Chinese Agricultural Education

ARM is a rather particular profession in China. Over the past four decades, China has experienced rapid urbanization. Due to the existence of the urban-rural binary system, China’s urban and rural areas are divided into two completely different labor markets (Liu, 2005; Meng, 2012) that imply two different social systems (Wu and Zhang, 2018; Xie et al., 2020). Most rural residents can only earn income from agricultural production. However, in China, the average income in the agricultural sector is much lower than that of the industrial sector found in cities. Moreover, there are less available social services in rural areas as compared to cities. The household registration system also limits rural residents’ access to public services by restricting their ability to move to urban areas. As a result, rural life in China often means low income and public welfare. Most rural young people expect to leave rural areas through higher education, as this is the easiest route to achieve a status change. For these reasons, ARM has never been a popular major in China, and most people have a strong bias against it.

Also, most young people do not like the teaching and training model of ARM. In China, ARM requires many experimental classes that do not take place entirely in the lab but need to be conducted in farmland far from the campus. This particular teaching model leads to less interaction between ARM students and NARM students and results in ARM students being frequently labeled as farmers. In the context of urbanization, the goal of most young Chinese is to pursue higher education in order to obtain a white-collar career and become middle-class. This can be seen as a lifestyle choice that offers the promise of a relatively stable and easy job (Wu et al., 2017). Despite a rapid increase in the social importance of agriculture (Eaglesham, 2006; Jordan et al., 2007; Meyer et al., 2008), there is widespread unwillingness among many students to choose agriculture as a major (Osborne and Dyer, 2000; Esters, 2007; Enayat and Naser, 2013). Due to the influence of social pressures, most ARM students in this study were not satisfied with their majors, and lacked a strong interest. They thus have been passive in academic performance and have experienced more significant stress than other students (Yueh et al., 2014). That is why ARM colleges always emphasize the need for students to “endure loneliness” or to “make sacrifices.”

Therefore, this paper focuses on the following two issues:

First, are there any significant differences in Students’ personality traits when they start school? Do these change after 3 years of study?
Second, why do changes in personality traits occur differently in students with different majors?

## Materials and Methods

### Data Source

Based on the *Ordinary Undergraduate Professional Directory (2012)* issued by China’s Ministry of Education, we categorized students whose majors were in agronomy, horticulture, veterinary medicine, plant protection, etc. as the ARM group, and those whose majors were in accounting, administration management, sociology, land resources management, etc. as the NARM group. We conducted three surveys among the ARM and NARM groups to compare differences in their personality traits.

Survey 1 was first conducted in university A (UA) in 2014, and our questionnaire surveyed 874 students. Among them, 384 were in the ARM group and 490 were in the NARM group. In 2017, we gave a second questionnaire to the 2014 respondents, of which a total of 660 were completed and returned; among them, 298 were from the ARM group, and 362 were from the NARM group (Table 1). The reliability coefficients for the 2014 and 2017 questionnaires were 0.806 and 0.792, respectively. The fact that both of these reliability coefficients were high (greater than 0.65), indicates that the study data was reliable. The KMO values were 0.748 and 0.675, respectively. Being greater than 0.5, this indicates that the questionnaire data was suitable for factor analysis. The *p*-value of Bartlett’s test was less than 0.05, which was considered valid for this questionnaire. The follow-up data from Survey 1 was designed to look for changes in personality traits between the two groups of students after 3 years of study in different disciplines.

**TABLE 1:** Descriptive statistics of the demographic variables of the sample in survey 1.

		AMR		NARM	
Time	Total	Male	Female	Male	Female
October, 2014	874 (100%)	148 (16.93%)	236 (27.00%)	218 (24.94%)	272 (31.12%)
October, 2017	660 (100%)	130 (19.70%)	168 (25.45%)	163 (24.70%)	199 (30.15%)

To further validate our findings, we also conducted Survey 2 and Survey 3 in 2018 (Table 2). Survey 2 was conducted at UA with respondents who were freshmen and seniors in ARM and NARM, respectively. Survey 3 was conducted in the same way as Survey 2, except that it was conducted at another top agricultural university in China, identified as university B (UB). This second university acted as an independent validation for the study. It is worth noting that, unlike Survey 1, Surveys 2 and 3 control for the gender of the respondents based on the overall male to female ratio of the school (2:3). In addition to the selection of respondents, university teachers were also interviewed in depth (Table 3). The same questionnaire was used in both surveys, and the results were integrated in order to perform reliability and validity tests. These results showed that the reliability coefficient was 0.670, well within the acceptable range, indicating that the research data was reliable. The KMO value was 0.865, which, being greater than 0.5, indicates that the questionnaire data was suitable for factor analysis. The *p*-value of Bartlett’s test was less than 0.05, and the questionnaire was considered valid. The interview material is mainly addressed in the “Discussion” section.

**TABLE 2:** Descriptive statistics of the demographic variables of the sample in Survey 2 and 3.

			ARM		NARM	
Time	University		Male	Female	Male	Female
Oct, 2018	University A	Freshman	40	60	40	60
		Senior	40	60	40	60
Oct, 2018	University B	Freshman	40	60	40	60
		Senior	40	60	40	60

**TABLE 3:** List of interviews conducted between 2017 and 2018.

No.	Venues	Date	Interviewees	Content
1–6	Meeting room	July 2017	ARM student	Knowledge and preference of the profession
7–12	Meeting room	July 2017	NARM student	Knowledge and preference of the profession
13–15	Office	January 2018	ARM teacher	Teaching style and student management
16–21	Office	January 2018	NARM teacher	Teaching style and student management

### Research Design

The Sixteen Personality Factor Questionnaire (16PF) and the NEO Five-Factor Inventory (NEO-FFI) were the best accepted and the most commonly used measures of personality traits, and they both have been extensively researched (Edward and Patrick, 2004; Zhang et al., 2013; Ole and Torill, 2015). 16PF and NEO-FFI inventories roughly measure the same aspects of personality (Rossier et al., 2004). The use of two measures of personality traits facilitates both the cross-validation, and the mutual validation of findings (Mani et al., 2013). In this paper, a follow-up survey (from freshman to junior year) was done for ARM and NARM students using the 16PF. Then, personality traits were tested using the NEO-FFI for freshmen and seniors in the ARM and NARM groups. The first group’s follow-up survey provides a perspective on student growth. We first tested whether, among students with different majors, there were already significant differences in their personality traits when they entered school in their freshman year. We then examined how their personality traits changed after 3 years (junior year). The latter two sets of surveys portray two cross-sections of personality traits for freshman and senior ARM and NARM students, respectively.

The personality traits test used in Survey 1 was the fifth edition of the 16PF questionnaire (Cattell and Schuerger, 2003), consisting of 185 questions that measure 16 primary factors. Specifically, the metrics measured include warmth, reasoning, emotional stability, dominance, liveliness, rule-consciousness, social boldness, sensitivity, vigilance, abstractedness, privateness, apprehension, openness to change, self-reliance, perfectionism, and tension (Cattell, 1989; Russell and Karol, 1994). Each scale ranges from 1 to 10, with a mean of 5.5, and a standard deviation (SD) of 2.

The NEO-FFI was used in Surveys 2 and 3. NEO-FFI is one of the most frequently used instruments in the evaluation of the Big Five factors: neuroticism, extraversion, openness, agreeableness, and conscientiousness. NEO-FFI consists of 60 questions with a 5-Likert scale response format (0–4 points), which measures the five factors mentioned above. The scales are bipolar—that is, each end of the scale has a distinct definition and meaning. High and low scores are regarded as neither good nor bad. The two questionnaires are used in order to avoid errors that could be caused by a single measurement.

Questionnaires on the Internet can be judged logically to avoid phenomena that tend to occur in traditional questionnaires; for example, answering questions randomly or omitting specific questions (Rice et al., 2017). Both the 16PF and NEO-FFI questionnaires were thus completed online by a sample of selected respondents, and results were downloaded from the website. Scores from each of the measures were computed and entered into SPSS. One-way analysis of variance (ANOVA) and “Cohen’s d” were also used in each survey. Cohen (1988, 1992) has made some widely used suggestions about what constitutes a large or small effect size: *d* = 0.2 (small), 0.5 (medium), and 0.8 (large).

## Results

### Survey 1: Panel Study Using 16PF at UA

A randomly selected group of freshmen was invited to UA in October 2014 and given a 16PF questionnaire. A total of 874 freshmen from five faculties (the Faculties of Plant Science, Animal Science, Biology and Environment, Food and Engineering, and Social Science) participated in the survey, with 384 (43.94%) students in the ARM group and 490 (56.06%) students in the NARM group. Among them 508 (58.12%) were female and 366 (41.88%) were male. All 874 responses were valid. In 2017, the same participants were invited to complete the same questionnaire again. A total of 362 (54.85%) from the NARM group and 298 (45.15%) from the ARM group completed the questionnaire, with 367 (55.60%) female students and 293 (44.40%) male students (Table 1). There were 660 valid responses. The questionnaire was available to the participants online for 2 weeks in both 2014 and 2017.

Table 4 shows results for means, SD, one-way analysis of variance (ANOVA), and analysis of “Cohen’s d” in the ARM and NARM groups for each of the personality factors in 2014 and 2017. From the results, we found no significant differences in the 16 personality factors between the two groups in 2014, and all had tiny effect sizes. However, the results in 2017 indicate that there are several differences between the means of the ARM and NARM on personality factors: the NARM were higher than the ARM in the sten scores of A (Warmth), E (Dominance), and H (Social Boldness), while the NARM were lower than the ARM in Q2 (Self-Reliance). We found some significant correlations in Warmth (*d* = 0.21), Dominance (*d* = 0.24), and Social Boldness (*d* = 0.30), with small to medium effect sizes.

**TABLE 4:** Means, SD, and Cohen’s d on the 16PF for the ARM and NARM between 2014 and 2017.

	2014						2017					
	ARM		NARM				ARM		NARM			
16 Personality factors	Mean	*SD*	Mean	*SD*	*p*	*d*	Mean	*SD*	Mean	*SD*	*p*	*d*
A (Warmth)	5.612	1.167	5.678	1.159	0.408	0.06	5.480	2.030	5.898	1.896	0.007**	0.21
B (Reasoning)	4.813	1.275	4.837	1.297	0.782	0.02	5.168	1.873	5.298	1.926	0.381	0.07
C (Emotional Stability)	5.151	1.296	5.088	1.310	0.477	−0.05	5.242	1.664	5.008	1.682	0.075	−0.14
E (Dominance)	5.466	1.304	5.322	1.267	0.101	−0.11	6.010	1.772	6.442	1.868	0.003**	0.24
F (Liveliness)	4.854	1.541	4.773	1.623	0.456	−0.05	6.960	2.262	6.956	2.228	0.982	0.00
G (Rule-Consciousness)	3.023	1.219	2.871	1.173	0.062	−0.13	4.705	1.651	4.713	1.790	0.953	0.00
H (Social Boldness)	7.443	1.102	7.500	1.065	0.437	0.05	5.594	1.803	6.135	1.787	0.000**	0.30
I (Sensitivity)	5.747	1.626	5.833	1.649	0.446	0.05	4.872	1.836	5.141	1.852	0.063	0.15
L (Vigilance)	6.221	1.499	6.198	1.474	0.817	−0.02	4.879	1.816	4.950	1.816	0.617	0.04
M (Abstractedness)	4.992	1.477	4.920	1.556	0.489	−0.05	5.789	1.683	5.646	1.784	0.296	−0.08
N (Privateness)	4.359	1.629	4.204	1.669	0.168	−0.09	6.513	1.619	6.436	1.642	0.547	−0.05
O (Apprehension)	4.966	1.065	5.055	1.208	0.256	0.08	5.933	1.932	5.854	1.929	0.600	−0.04
Q1 (Openness to Change)	5.698	1.918	5.678	1.917	0.876	−0.01	5.826	1.753	5.972	1.780	0.289	0.08
Q2 (Self-Reliance)	3.133	1.418	3.163	1.529	0.763	0.02	5.359	1.949	5.039	1.811	0.029*	−0.17
Q3 (Perfectionism)	3.719	1.722	3.659	1.665	0.605	−0.04	5.302	1.656	5.218	1.662	0.519	−0.05
Q4 (Tension)	6.279	1.269	6.371	1.331	0.297	0.07	5.785	1.570	5.812	1.724	0.835	0.02

*p < 0.05; **p < 0.01.

It can be inferred that differences occur in the personality traits between ARM and NARM groups after 3 years of discipline-related education. According to the essentials of the 16PF assessment (Cattell and Schuerger, 2003), the ARM students tend to be socially timid and not good at expressing themselves. They prefer keeping a certain emotional distance between themselves and others, and when compared with the NARM, are more interested in working or solving problems alone. The NARM group tends to have a genuine interest in people, and they find interacting with others intrinsically rewarding. This echoes what Suvedi et al. (2016) have found; i.e., students at agricultural colleges have a lower disposition for verbal communication skills.

### Survey 2: Verification Study Using NEO-FFI at UA

Survey 2 was also conducted at UA to verify the results from Survey 1. Setting the male-to-female sample ratio to be the same as that in Survey 1, we then randomly invited 100 freshmen and 100 seniors from ARM and NARM to complete the NEO-FFI online questionnaire in October 2018, ensuring there were 40 males and 60 females in each group (Table 2).

As Table 2 shows, there is no significant difference in the five personality dimensions between the two groups’ freshmen, and all showed a low effect size. However, there were differences among the seniors (Table 5). A highly statistically significant result appeared for N (Neuroticism) when the ARM (mean: 24.52; SD: 5.51) and NARM (mean: 22.16; SD: 5.76) were compared at the 1% level. Also, E (Extraversion) produced a significant statistical difference between the ARM (mean: 24.11; SD: 5.89) and NARM (mean: 26.01; SD: 6.39) (*p* < 0.05). At the same time, a small effect size was found for C (Conscientiousness), while N (Neuroticism) and E (Extraversion) showed small to medium effect sizes.

**TABLE 5:** Means, SD and Cohen’s d on the **NEO-FFI** for the ARM and NARM in freshmen and seniors in 2018 (University A).

	Freshman						Senior					
	ARM		NARM				ARM		NARM			
NEO-FFI	Mean	*SD*	Mean	*SD*	*p*	*d*	Mean	*SD*	Mean	*SD*	*p*	*d*
N (Neuroticism)	22.24	7.64	21.97	6.21	0.784	−0.04	24.52	5.51	22.16	5.76	0.003**	−0.42
E (Extraversion)	26.41	6.13	26.73	6.16	0.713	0.05	24.11	5.89	26.01	6.39	0.030*	0.31
O (Openness)	27.24	5.27	26.47	5.42	0.310	−0.14	25.63	4.92	26.45	5.06	0.247	0.16
A (Agreeableness)	27.86	4.40	28.63	4.39	0.217	0.18	28.17	3.86	28.62	4.15	0.429	0.11
C (Conscientiousness)	29.12	7.40	29.45	6.70	0.741	0.05	28.04	5.28	29.07	5.06	0.161	0.20

*p < 0.05; **p < 0.01.

According to the essentials of the NEO-FFI, neuroticism measures emotional instability. This indicates that when compared to the NARM student, the ARM student tends to get worried and anxious more easily, and does not cope effectively with stress. Extraversion is defined as an individual’s tendency to be outgoing, sociable, and communicative rather than being reserved. The results indicate that the NARM seniors are better at expressing themselves and communicating with others than the ARM seniors. There was no significant difference in the five personality dimensions between the two groups’ freshmen. This is also consistent with the results of Survey 1.

### Survey 3: Verification Study at UB Using NEO-FFI

To test the results of Survey 2, we repeated it at UB. In October 2018, 100 freshmen and 100 seniors from ARM and NARM were randomly invited to finish the NEO-FFI questionnaire online. Of these, 40% were males and 60% were females (Table 2).

The data (Table 6) indicates that, with regards to the NEO-FFI dimensions in the two groups’ freshmen, there were no significant differences and universally low effect sizes. However, we observed significant differences between the ARM and the NARM seniors in terms of E (Extraversion) (ARM mean: 25.87, SD: 4.61 vs. NARM mean: 27.31, SD: 4.41; *p* < 0.05) and C (Conscientiousness) (ARM mean: 31.24, SD: 5.98 vs. NARM mean: 28.96, SD: 5.27; *p* < 0.01). The E (Extraversion), O (Openness), A (Agreeableness) and C (Conscientiousness) dimensions all indicated a small to medium effect size.

**TABLE 6:** Means, SD and Cohen’s d on the **NEO-FFI** for the ARM and NARM in freshmen and seniors in 2018 (University B).

	Freshman						Senior					
	ARM		NARM				ARM		NARM			
NEO-FFI	Mean	*SD*	Mean	*SD*	*p*	*d*	Mean	SD	Mean	*SD*	*p*	*d*
N (Neuroticism)	22.19	6.29	22.48	5.90	0.737	0.05	19.95	7.17	20.48	5.34	0.554	0.08
E (Extraversion)	24.36	5.12	23.86	5.99	0.526	−0.09	25.87	4.61	27.31	4.41	0.025*	0.32
O (Openness)	28.88	4.83	28.35	4.56	0.426	−0.11	25.96	4.42	27.15	4.66	0.065	0.26
A (Agreeableness)	27.84	4.79	28.13	3.32	0.619	0.07	29.05	3.39	28.17	4.49	0.119	−0.22
C (Conscientiousness)	29.66	6.76	30.38	5.60	0.413	0.12	31.24	5.98	28.96	5.27	0.005**	−0.40

*p < 0.05; **p < 0.01.

Both Survey 2 and Survey 3 show that the average self-reported level of E (Extraversion) in the NARM group was higher than that of the ARM group (*p* < 0.05). We also found differences in the factors between the two universities. The average score for N (Neuroticism) from the ARM group at UB was relatively higher than that of the NARM group at UA, while the average score for C (Conscientiousness) from the ARM group at UA was higher than that of the NARM group at UB.

The results of Survey 3 tell us that the difference found at UA also exists at a second agricultural university (UB). This supports the conclusions we drew from Survey 1; i.e., differences develop between the employability and personality traits of the two student groups after 3 years of discipline-related courses. In turn, this validates the hypothesis that the particular area of study a student chooses may affect the development of their personality traits and employability.

## Discussion

In both time and resources, education is one of the most prolonged and intense periods in which society influences psychological functioning (Joshua, 2011). The college years are often thought of as a time of personal transformation. As mentioned above, individuals become more independent, explore new opportunities, and reconsider their values, goals, and self-beliefs (Robins et al., 2005). Educational experiences impart skills that adhere to particular personality traits, thus influencing employability (Heckman et al., 2010). The present study examines the differences between the personality traits of students in the two groups, and investigates the impact of particular disciplines on those personality traits. The three surveys indicated that courses of study do affect the development of personality traits in students. To better understand how the relevant areas of study affect personality development, we introduce the socio-economic model of personality traits proposed by Roberts and Jackson (2008), which describes how experiences change personality traits. According to this model, environmental experiences do not directly affect personality traits; they can only affect personality traits when changes in state exist for a prolonged period of time (Roberts and Jackson, 2008). Therefore, it is believed that a change in personality traits occurs through a relatively consistent experience that leads to lasting changes in the way one behaves, thinks, or feels. This is consistent with the findings of Kohn and Schooler (1978) who concluded that if one’s behaviors remain distinct from others for an extended period of time, these behaviors will become internalized, leading to changes in personality traits. In other words, differences in personality traits are more likely to occur if one group of people has a relatively consistent experience that differs from another group.

### Specific Curricula Lead to Differences in Personality Traits

With the socio-economic model in mind, we compared the two groups’ educational objectives and areas of study at UA and found several significant differences (Table 7). ARM students pay more attention to improving their academic capabilities than do NARM students, but lack teamwork, self-display, and communicative skills. NARM students, on the other hand, focus on the enhancement of comprehension and expressiveness.

**TABLE 7:** The comparison in discipline courses between ARM and NARM at UA.

		ARM	NARM
Proportion of experimental classes in discipline courses		20.89%	3.80%
Minimum credits required to complete	Theoretical learning	63 credits	75 credits
	Experimental learning	12 credits	0 credit
	Practical learning	20 credits	20 credits
Representative courses		Inorganic and analytical chemistry	Principles of management
		Organic chemistry	Principles of economics
		Physics	Introduction of sociology
		Botany	Human resource management
		Zoology	Probability theory and mathematical statistics
		Microbiology	Calculus
		Biostatistics and experimental design	Macroeconomics
		General introduction to soil-fertilizer science	Microeconomics

This phenomenon can be explained by the significant differences between the two groups in terms of course types and credits. Both groups have their discipline-specific courses. The ARM group’s representative courses are inorganic and analytical chemistry, organic chemistry, physics, botany, zoology, and microbiology; all of these courses require a lot of experiments to verify theories^1^. On the other hand, the representative courses of the NARM group are principles of management, principles of economics, introduction to sociology, and human resource management; all courses for which academic assignments usually consist of surveys and reports. Moreover, experimental courses account for 20.89% of the total number of ARM courses, while in the NARM group that ratio is only 3.8%. The NARM group needs to take theory courses totaling 75 credits, while the ARM only takes 63 credits. Also, the ARM group must take experimental courses totaling at least 12 credits, while the NARM group is not required to take any such courses. The survey at UB also confirmed this.

We also interviewed some teachers to further clarify the differences in the areas of study undertaken by the two groups. The results confirm that most of the course assignments for the ARM group are small-scale experiments in the lab, which require the students to think and solve problems independently. The NARM course assignments are usually surveys and reports, which require the students to form groups and present their research findings as a team. As one ARM teacher put it:

*[*…*] There is no clear guide for NARM students, and students can learn what they want to from different teachers. Although ARM students similarly do not have a clear guide, the major requires them to be in a lab and follow a particular research team. This, in a way, determines the direction of their future research. (interviewed by authors in Jan. 2018, see Table 3, No.13).*

NARM students do not have these limitations; they continue to focus on more basic expertise during their college years. Further clarification of their professional direction comes at the graduate level. At the same time, after their sophomore year, the survey-based professional teaching model gives them more opportunities to interact with others. As one NARM teacher stated:

*[*…*] Our major places a lot of emphasis on teamwork, and if you can’t work well with your classmates, it will be challenging to work with other colleagues in the workplace later on. (interviewed by authors in Jan. 2018, see Table 3, No.20).*

This difference in course assignments, in turn, led to differences in thinking and behaving by the end of the 3 year discipline-specific education, thereby supporting the claim that a relatively consistent experience leads to lasting changes in the way one behaves, thinks, or feels.

### Social Bias Creates Increased Differentiation in Personality Traits

One of the purposes of an agricultural education at the university level is to prepare young people and adults for careers in the field of agriculture (Westbrook et al., 2008; Goecker et al., 2014). However, Chinese people have been deeply inculcated with the idea that agricultural jobs are of low status. This social bias undoubtedly also widens the differences in personality traits between ARM and NARM students. Our survey found that ARM students do hold their major in higher regard than other majors. Many Students’ first choice before entering the school is not ARM; however, other majors have limited enrollment and these students are simply assigned to ARM by the school. Therefore, many students fight for the opportunity to change their majors in their sophomore year; as one ARM teacher said:

*[*…*] The big challenge for ARM is when students are triaged in their sophomore year. Although some students from other majors transfer to ARM, more students are transferring away than moving in. (interviewed by authors in Jan. 2018, see Table 3, No.15).*

Secondly, social prejudice against ARMs inevitably permeates the campus as well. ARM students spend a large part of their time in the laboratory – no different from other natural disciplines. However, NARM students will always equate ARM with farming. As some ARM students put it:

*[*…*] When my other high school classmates found out about my major, they would always ask, “Is this farming?” or “Is farming hard?” (interviewed by authors in Jul. 2017, see Table 3, No.5).*

The societal bias against ARM that permeates the campus also makes ARM students less willing to interact with students from other majors; ARM students tend to interact more with students or teachers. In this way, the social circle of ARM students is narrower than that of NARM students.

## Suggestions for Optimizing Chinese ARM Education

As personality may affect employability, this study provides several implications for student support at agricultural universities. ARM education may include more public-speaking or communication-intensive modules or components. For example, students usually follow the teachers’ instructions and duplicate the experimental process individually when taking practical courses. This is more imitation than creation. They finish the assignments independently rather than by teamwork. Such courses could be improved by including collaborative design, and the conducting and analyzing of experiments; thus giving students more opportunities to discuss how to design experiments, carry them out, and analyze the data they yield. ARM students should also be encouraged to participate in extracurricular activities and join student clubs where they can work with peers. For example, they could take part in social service activities that apply disciplinary knowledge, or attend regular academic seminars to share their learning and experiences with others. Exposure to agriculture-related businesses and personnel would also help ARM students to network; providing them with opportunities such as internships at internationally renowned agricultural companies, on-campus activities involving successful practitioners of agriculture, and alumni meetings. The university should also work with the government to remove social prejudice against ARM, even in agriculture. New programs or concentrations combining traditional agriculture, modern technology, and the wellbeing of humanity are suggested options. This could offer a broader range of career opportunities, fostering more positive attitudes toward agriculture among the students.

## Conclusion

Our research captures the impact of academic disciplines on the formation of personality traits. Particular disciplines have a slight strengthening effect on Students’ personality traits, affecting their employability. Thus, this study demonstrates that a Student’s chosen area of study impacts their personality development. The current study has presented useful information and research regarding the impact of academic disciplines on personality formation. It also suggests new interdisciplinary programs and various activities that are meant to improve communication skills among ARM students while elevating the perception of ARM. It is hoped that the present study will stimulate further exploration into how specific disciplines are related to changes in a particular personality trait. Of course, this study has some shortcomings that can be used as a direction for further in-depth research in the future. For example, gender and the subtle effects of different instructors on the formation of Students’ personality traits are not addressed in this paper.

## Data Availability Statement

The raw data supporting the conclusions of this article will be made available by the authors, without undue reservation.

## Author Contributions

All authors listed have made a substantial, direct and intellectual contribution to the work, and approved it for publication.

## Conflict of Interest

The authors declare that the research was conducted in the absence of any commercial or financial relationships that could be construed as a potential conflict of interest.

## Footnotes

1 There are usually three types of courses in Chinese universities; namely, theoretical courses, experimental courses, and practicums.
